# Critical Role of the Virus-Encoded MicroRNA-155 Ortholog in the Induction of Marek's Disease Lymphomas

**DOI:** 10.1371/journal.ppat.1001305

**Published:** 2011-02-24

**Authors:** Yuguang Zhao, Hongtao Xu, Yongxiu Yao, Lorraine P. Smith, Lydia Kgosana, James Green, Lawrence Petherbridge, Susan J. Baigent, Venugopal Nair

**Affiliations:** 1 The Division of Structural Biology, The Wellcome Trust Centre for Human Genetics, Oxford University, Oxford, United Kingdom; 2 Avian Oncogenic Virus Group, Avian Infectious Disease Programme, Institute for Animal Health, Berkshire, United Kingdom; Duke University Medical Center, United States of America

## Abstract

Notwithstanding the well-characterised roles of a number of oncogenes in neoplastic transformation, microRNAs (miRNAs) are increasingly implicated in several human cancers. Discovery of miRNAs in several oncogenic herpesviruses such as KSHV has further highlighted the potential of virus-encoded miRNAs to contribute to their oncogenic capabilities. Nevertheless, despite the identification of several possible cancer-related genes as their targets, the direct *in vivo* role of virus-encoded miRNAs in neoplastic diseases such as those induced by KSHV is difficult to demonstrate in the absence of suitable models. However, excellent natural disease models of rapid-onset Marek's disease (MD) lymphomas in chickens allow examination of the oncogenic potential of virus-encoded miRNAs. Using viruses modified by reverse genetics of the infectious BAC clone of the oncogenic RB-1B strain of MDV, we show that the deletion of the six-miRNA cluster 1 from the viral genome abolished the oncogenicity of the virus. This loss of oncogenicity appeared to be primarily due to the single miRNA within the cluster, miR-M4, the ortholog of cellular miR-155, since its deletion or a 2-nucleotide mutation within its seed region was sufficient to inhibit the induction of lymphomas. The definitive role of this miR-155 ortholog in oncogenicity was further confirmed by the rescue of oncogenic phenotype by revertant viruses that expressed either the miR-M4 or the cellular homolog gga-miR-155. This is the first demonstration of the direct *in vivo* role of a virus-encoded miRNA in inducing tumors in a natural infection model. Furthermore, the use of viruses deleted in miRNAs as effective vaccines against virulent MDV challenge, enables the prospects of generating genetically defined attenuated vaccines.

## Author Summary

MicroRNAs (miRNAs), encoded in the genomes of a number of organisms including several viruses, belong to a class of small RNA molecules that can function as key regulators of gene expression influencing various biological processes and diseases including cancer. Among all the miRNAs, miR-155 has been well documented for its direct role of oncogenesis in a number of species including chickens. Remarkably, miR-K12-11 and miR-M4, the miRNAs encoded by the oncogenic Kaposi's sarcoma-associated herpesvirus (KSHV) and Marek's disease virus (MDV) respectively, have been shown to be functional orthologs of miR-155. There are no animal models of KSHV-induced tumors to examine the oncogenic potential of miR-K12-11. However, using recombinant mutant viruses in excellent models of MDV-induced lymphomas in their natural chicken hosts, we demonstrate that miR-M4 is critical for the induction of tumors. This is the first study that clearly demonstrates a direct role for a single miRNA in inducing cancer in an *in vivo* animal model. The ability of gga-miR-155 to rescue the oncogenic potential of miR-M4-deleted virus demonstrated the conservation of oncogenic functions the two miRNAs. Moreover, we show that virus attenuated by deleting the miRNAs can function as vaccines against virulent virus infection, enabling the prospects of generating novel molecularly-defined vaccines.

## Introduction

Oncogenic viruses account for nearly a fifth of all human cancers [1]. In addition to their devastating effects on human health, virus-induced neoplastic diseases are valuable models in understanding the molecular pathways and dynamics of cancer. Although most of the past studies on the molecular mechanisms of cancer induced by oncogenic viruses have primarily centred on classic oncogenes [2], increasing demonstration of the widespread role of micro(mi)RNAs in cancer has added new dimensions to the molecular mechanisms of neoplastic transformation [3], [4]. Moreover, the identification of miRNAs encoded by human oncogenic viruses such as Kaposi's sarcoma-associated herpesvirus (KSHV) and Epstein-Barr virus (EBV) suggested that virus-encoded miRNAs may also contribute towards the oncogenicity of the virus. The potential of some of these miRNAs to target a number of host genes associated with the oncogenic pathways has further strengthened the case for their role in inducing malignancies [5], [6]. However, despite the identification of several potential targets [7], direct *in vivo* role of virus-encoded miRNAs in the induction of malignancies is yet to be demonstrated, due at least in part, to the absence of suitable animal disease models.

Marek's disease (MD), a naturally occurring neoplastic disease of poultry [8], is an excellent model for herpesvirus-induced lymphomas [9], [10], [11], [12]. All birds exposed to the causative Marek's disease virus (MDV), unless protected by vaccination or have genetic resistance to the disease, develop rapid-onset T-cell lymphomas usually from 3–4 weeks after infection. Among the virus-encoded genes, Meq protein encoded from the MDV E *co*RI Q fragment is considered to be primarily associated with the oncogenicity of the virus, since its deletion [13] or inhibition of its interaction with host proteins such as c-Jun, c-Fos and C-terminal binding protein [11], [14], [15] can abolish the oncogenic potential of the virus. Although these studies clearly demonstrate the contribution of Meq in oncogenesis, other viral genes could also be important in the induction of MD lymphomas. We and others have shown that MDV also encodes 14 miRNAs located within three clusters from each of the long (IR_L_/TR_L_) and short (IR_S_/TR_S_) repeat regions of the viral genome [16], [17]. Most of these miRNAs are expressed at high levels in MD lymphomas and transformed T-cell lines [18] suggesting their direct role in transformation. The six miRNAs in cluster 1, thought to be processed from a single primary transcript upstream of the *Meq* locus [16] included miR-M4, the functional ortholog of miR-155 [19] and KSHV-miR-K12-11 [20], [21]. MDV miR-M4 has recently been shown to inhibit the translation of viral proteins involved in the cleavage and packaging of herpesvirus DNA [22]. However, based on the direct association of miR-155 in several cancers [23], [24], including the recent findings on its roles in TGF-β pathway and lymphomagenesis [25] and modulation of mismatch repair and genomic instability [26], we hypothesised that miR-M4 has a major role in MDV oncogenicity. This was also supported by recent observations of high levels of its expression in tumors correlated with the pathogenicity of viral strains [17].

In the present study, we explored the functional role of the miRNAs encoded within the cluster 1 of the MDV genome, including that of the miR-155 functional ortholog miR-M4, in inducing T-cell lymphomas using the well-established models of MD in the natural chicken hosts of the virus. Using a series of mutant viruses generated by reverse genetics techniques [27], [28] on the full-length infectious bacterial artificial chromosome (BAC) clone of the highly oncogenic RB-1B virus [29], [30], we were able to directly examine the *in vivo* functions of MDV-encoded miRNAs in the induction of MD lymphomas. Our studies demonstrate the critical role of the cluster 1 miRNAs, especially of the miR-M4, in the induction of lymphomas, thus providing the direct evidence on the *in vivo* function of miRNAs in virus-induced cancer.

## Results

### Cluster 1 miRNAs are not essential for MDV replication

**Figure 1:**
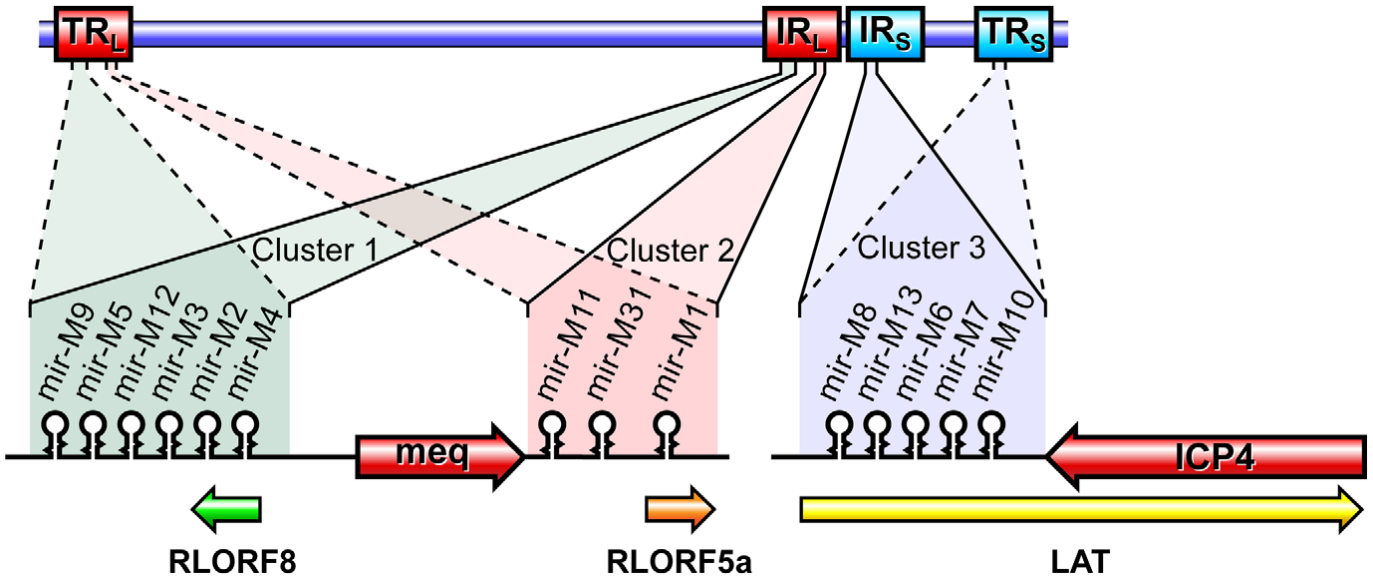
Genome structure of MDV showing positions of the miRNAs. Schematic illustration of the MDV genome structure with the TR_L_ (terminal repeat long), IR_L_ (internal repeat long), TR_S_ (terminal repeat short) and IR_S_ (internal repeat short) regions, with the positions of the miRNAs encoded within the 3 clusters. The position and the orientation of the viral genes RLORF8, Meq, RLORF5a, LAT and ICP4 that overlap the miRNA loci are indicated. Identical copies of the miRNAs in two orientations in the terminal and internal repeat regions are indicated in continuous and dotted lines.

Members of the Family *Herpesviridae* account for most of the currently identified virus-encoded miRNAs, and these are thought to play important roles in the distinct biological and pathogenic features of these viruses. MDV encoded-miRNAs are expressed as 3 clusters from the repeat regions, making each miRNA present as two identical copies in the viral genome (Fig. 1). We have previously demonstrated high levels of expression of these miRNAs both *in vitro* and *in vivo* [16], [31]. However, these studies did not show whether any of these miRNAs are essential for viral replication. In order to address this, we examined the role of cluster 1 miRNAs using a series of mutant viruses generated by reverse genetics techniques on the pRB-1B5 BAC clone [29]. In the mutant virus construct miR-00, the region encoding the miRNA cluster and the overlapping R-LORF8 in the TR_L_ or IR_L_ regions were deleted by recombination using *gal*K-kan and *thy*A-spec antibiotic selection cassettes. While *gal*K-kan cassette replaced the entire region encoding all the six miRNAs from one of the repeat regions, *thy*A-spec cassette that targeted a shorter region removed all the miRNAs except miR-M9 and miR-M4 from the other repeat region.

**Figure 2:**
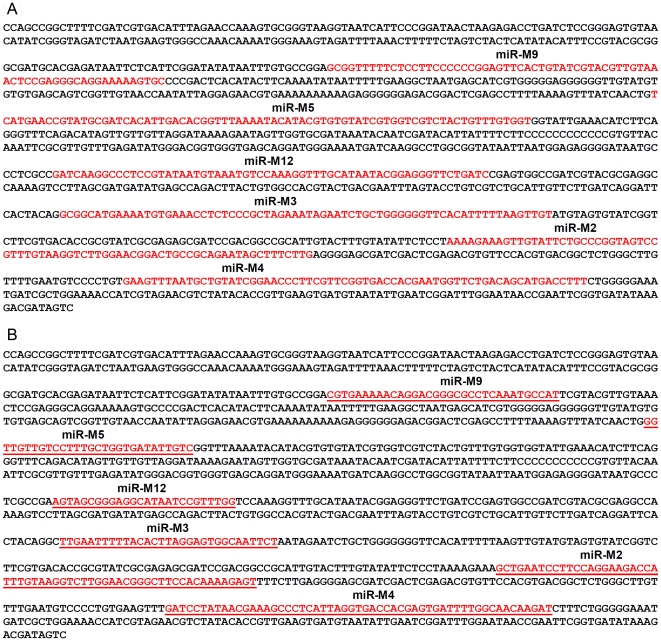
Mutations in the sequences of the miRNAs in the cluster. (a) The sequence of the cluster 1 miRNA locus (nucleotides 134782–136187) in the MDV genome from the pRB-1B5 virus (GenBank Accession EF523390) showing the positions of the pre-miRNA sequence of the 6 miRNAs (shown in red) encoded within this region. (b) Sequence of the synthetic gene of the corresponding region with mutated sequences of each of the miRNAs (shown in red and underlined). Mutations were chosen not to alter the predicted open reading frame of the R-LORF8 gene that overlaps this region.

**Figure 3:**
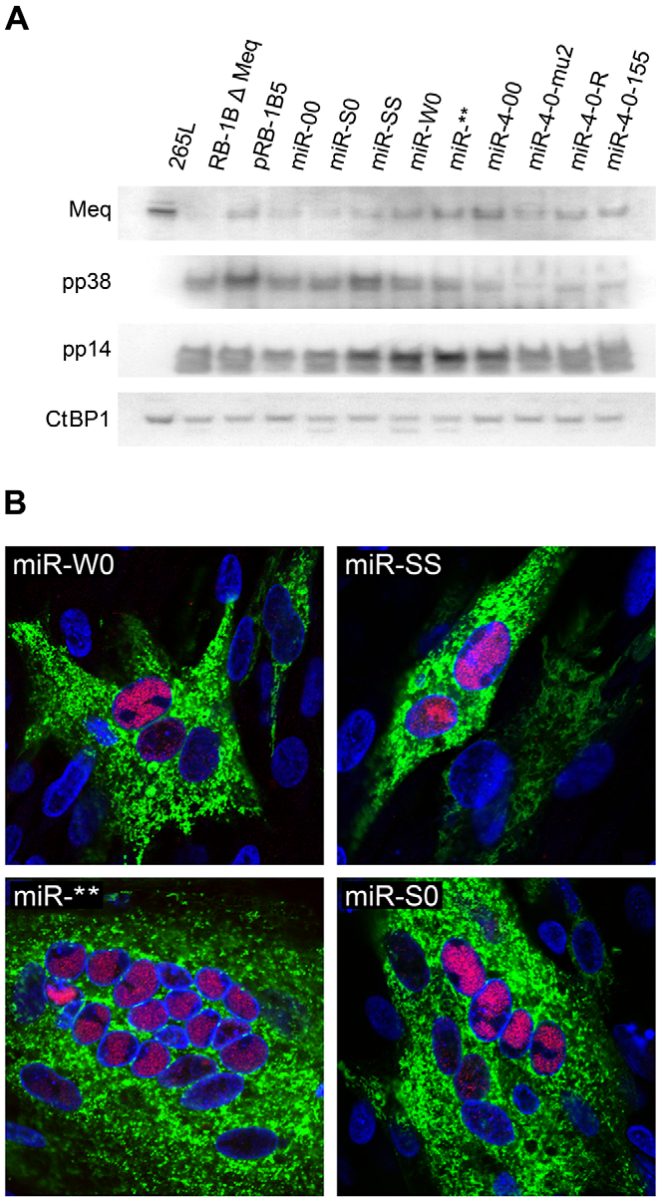
Expression of viral proteins by the mutant viruses. **A**) Western blotting with antibodies against viral antigens Meq (FD7), pp38 (BD1) and pp14 [46] on lysates of CEF infected with the parent pRB-1B5 and the different mutant viruses. MDV-transformed lymphoblastoid cell line 265L and Meq-deletion mutant RB-1BΔMeq were used as controls. Chicken CtBP1 [11] was used as the loading control. **B**) Immunofluorescence assay demonstrating the localisation of Meq (red) in the nucleus (DAPI-stained nucleus shown in blue) and pp38 (green) in the cytoplasm of CEF on glass cover slips 72 hours after infection with miR-W0, miR-SS, miR** and miR-S0 viruses.

**Figure 4:**
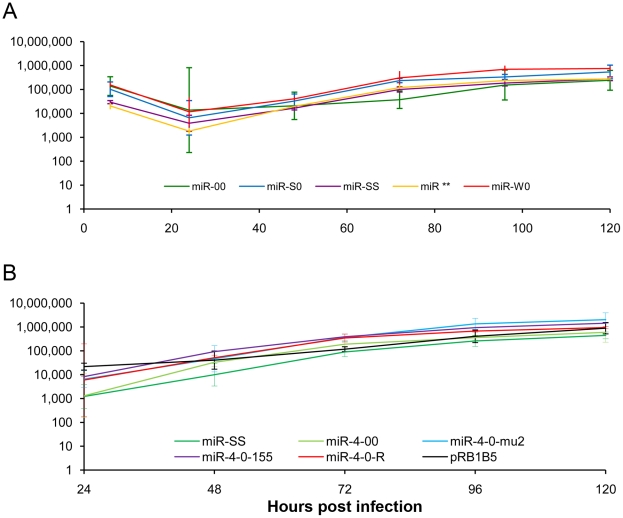
Comparison of the growth kinetics of different viruses. *In vitro* growth rates of viruses with mutations in the (**A**) miRNA cluster 1 and (**B**) miR-M4 locus measured from the viral genome copy numbers determined using TaqMan real-time qPCR on DNA extracted from CEF harvested at various time points after inoculation. Viral genome copy numbers per 10 000 cells (shown with 95% confidence intervals) are shown on the y axis.

**Figure 5:**
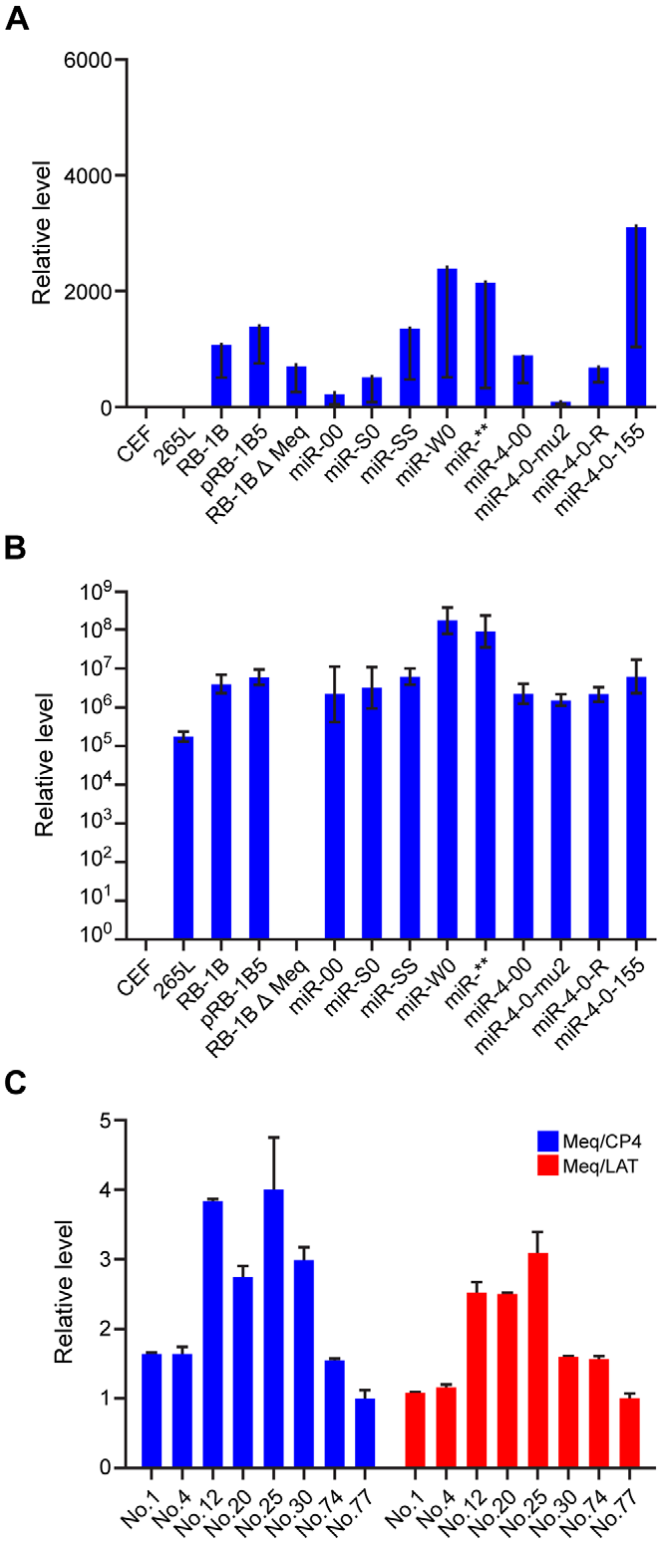
MDV gene expression in infected cells. Relative expression of MDV gene ICP4 (**A**) and Meq (**B**) measured by qRT-PCR (normalised to the GAPDH levels) in RNA extracted from CEF infected with the wild type RB-1B, the parent pRB-1B5 and the different mutant viruses. Uninfected CEF, MDV-transformed lymphoblastoid cell line 265L and Meq-deletion mutant RB-1BΔMeq were used as controls. (**C**) Relative expression levels of Meq, ICP4 and LAT in CEF co-cultured with the PBL collected from birds infected with different viruses. The relative expression levels of Meq normalised to ICP4 (blue) and LAT transcripts (red) are shown. Results represent mean of triplicate assays with error bars showing the standard errors of the mean. The identification number of individual birds infected with miR-SS (No. 1 & 4), miR** (No. 12 & 20), miR-W0 (No. 25 &30) and miR-S0 (No. 74 & 77) are shown.

For generation of viruses with mutations in all of the miRNAs within the cluster, we synthesised a gene designed to introduce mutations into the stem loop structures of each of the six miRNAs in the cluster 1 without affecting the R-LORF8 frame (Fig. 2). In the miR-S0 and the miR-SS constructs respectively, one or both copies of the miRNA locus were replaced with this synthetic gene sequence, preventing them expressing any of cluster 1 miRNAs. The revertant miR-W0 and miR** viruses both expressed all the miRNAs, but in the latter, the introduction of a stop codon is expected to abolish the translation of R-LORF8. These two constructs were included to delineate between the functions of miRNAs and the overlapping R-LORF8 gene. The details of mutations in each of the individual recombinant viruses are summarised in Table 1. Reconstitution of the viruses in primary chicken embryo fibroblast (CEF) transfected with the BAC DNA demonstrated the infectivity of each of the above constructs. Demonstration of viral proteins Meq, pp38 and pp14 in CEF infected with the parent pRB-1B5 and different mutant viruses by western blotting showed that the mutations did not affect the expression of these proteins (Fig. 3A). This was further confirmed by specific staining of Meq and pp38 in the nucleus and cytoplasm respectively of CEF infected with a selection of the mutant viruses (Fig. 3B). Moreover, analysis of the *in vitro* growth kinetics showed very similar replication kinetics for both the parent and mutant viruses (Fig. 4A) indicating that these miRNAs are not essential for replication of MDV *in vitro*. For further analysis of the effect of mutations in the miRNA cluster on MDV gene expression, we compared the relative levels of ICP4 and Meq transcripts by quantitative RT-PCR in CEF infected with the wild type RB-1B, parent pRB-1B5 and the mutant viruses. MDV-transformed lymphoblastoid cell line 265L and CEF infected with the Meq-deletion mutant (RB-1BΔMeq) virus were used as controls. These studies showed that the transcription of ICP4 and Meq were not affected by mutations in the miRNA locus (Fig. 5A–B), although the levels of ICP4 transcripts were low with variations among different viruses in comparison to the Meq transcripts.

**Table 1:** Profile of the expression of miRNAs and R-LORF8 in different constructs.

IR_L_								TR_L_						
Virus Constructs	M9	M5	M12	M3	M2	M4	R-LORF8	M9	M5	M12	M3	M2	M4	R-LORF8
miR-00	**−**	**−**	**−**	**−**	**−**	**−**	**−**	**+**	**−**	**−**	**−**	**−**	**+**	**−**
miR-S0	**−**	**−**	**−**	**−**	**−**	**−**	**+**	**−**	**−**	**−**	**−**	**−**	**−**	**−**
miR-SS	**−**	**−**	**−**	**−**	**−**	**−**	**+**	**−**	**−**	**−**	**−**	**−**	**−**	**+**
miR-W0	**+**	**+**	**+**	**+**	**+**	**+**	**+**	**−**	**−**	**−**	**−**	**−**	**−**	**−**
miR **	**+**	**+**	**+**	**+**	**+**	**+**	**−**	**+**	**+**	**+**	**+**	**+**	**+**	**−**
miR-4-00	**+**	**+**	**+**	**+**	**+**	**−**	**+**	**+**	**+**	**+**	**+**	**+**	**−**	**+**
miR-4-0-mu2	**+**	**+**	**+**	**+**	**+**	**−**	**+**	**+**	**+**	**+**	**+**	**+**	**−**	**+**
miR-4-0-155	**+**	**+**	**+**	**+**	**+**	**−** †	**+**	**+**	**+**	**+**	**+**	**+**	**−**	**+**
miR-4-0-R	**+**	**+**	**+**	**+**	**+**	**+**	**+**	**+**	**+**	**+**	**+**	**+**	**−**	**+**
pRB-1B5	**+**	**+**	**+**	**+**	**+**	**+**	**+**	**+**	**+**	**+**	**+**	**+**	**+**	**+**

**†:** Expresses one copy of miR-155.

In order to examine whether these miRNAs are essential for MDV replication *in vivo*, we asked whether viruses with mutations in the miRNA locus could be re-isolated from birds in later stages of infection. Successful isolation of MDV by co-culturing of the peripheral blood leukocytes (PBL) of birds infected with different viruses 7–8 weeks after infection confirmed that the mutations in the miRNA locus did not affect the viral replication and their ability to establish latent infection *in vivo*. Moreover, quantitative RT-PCR data of Meq, LAT and ICP4 transcripts in CEF co-cultured with the PBL from infected birds (Fig. 5C) indicated that the modifications in the miRNA locus did not affect the expression of these transcripts in viruses isolated from these birds. All these results confirmed that the miRNAs encoded from this locus did not affect MDV replication either *in vitro* or *in vivo*.

### Deletion of cluster 1 miRNAs abolishes oncogenicity of the virus

**Figure 6:**
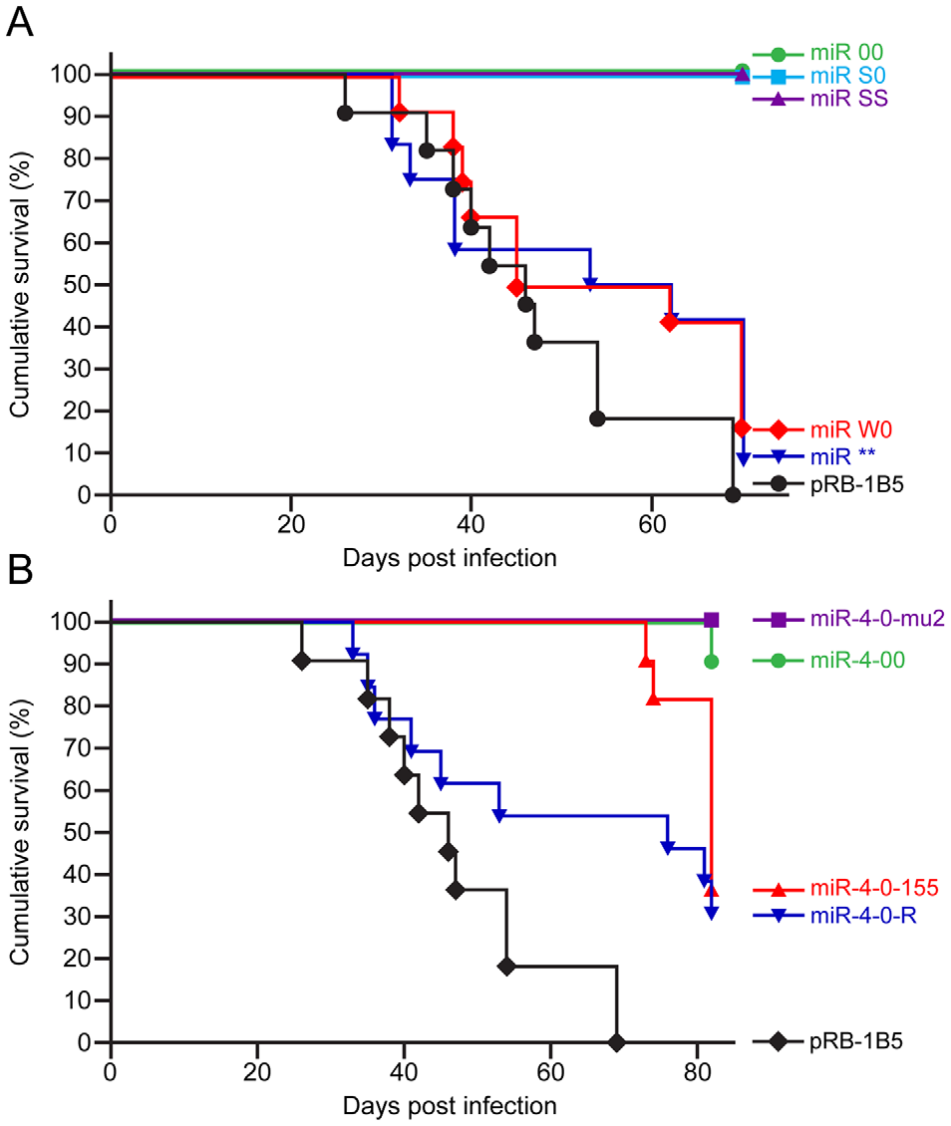
Incidence of MD in birds infected with recombinant viruses. Survival rates based on the morbidity and cumulative incidence of MD in SPF line P (B^21/21^) birds during the experimental period in groups of birds infected with (**A**) parent pRB-1B5 and mutant derivatives miR-00, miR-S0, miR-SS, miR-W0 and miR** and (**B**) parent pRB-1B5 and mutant derivatives miR-4-00, miR-4-0-mu2, miR-4-0-155 and miR-4-0-R. The incidence was determined from occurrence of MD determined at post mortem from gross or histopathological lesions of lymphomas.

Having shown that the mutations in the miRNA locus did not affect the replication and viral gene expression *in vitro* or the establishment of latency *in vivo*, we investigated whether the miRNAs encoded within this cluster are associated with the oncogenicity of the virus using the established infection models in the natural chicken hosts [29]. Compared to the 100 percent incidence of MD in birds infected with the parent pRB-1B5 virus (Fig. 6A), none of the birds infected with the miR-00, miR-S0 and miR-SS viruses developed any gross or microscopic lesions of MD, showing that the loss of expression of the cluster 1 miRNAs directly affected the oncogenic potential of MDV. Rescue of the oncogenic phenotype by the revertant miR-W0 virus to the levels close to that of the parent virus, even by restoring only one copy of the miRNA cluster, provided the strong evidence on the distinct role of these miRNAs in the induction of lymphomas. Moreover, the induction of MD in 90% of the birds by the miR** virus (Fig. 6A) demonstrated that miRNAs, but not R-LORF8, are involved in the induction of lymphomas.

### MDV miR-M4 is essential for oncogenicity

**Figure 7:**
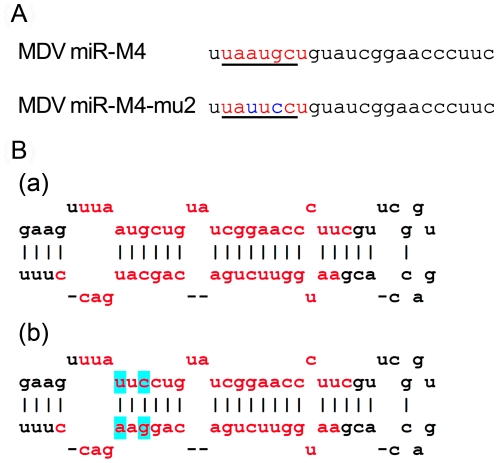
Sequence and structure of miR-M4 and the mutant region. (**A**) The sequence of the mature miR-M4 with the region of the seed region underlined and shown in red. The corresponding region of the miR-4-0-mu2 mutant, with the two nucleotide changes in the seed region is shown in blue. (**B**) The predicted stem loop structure of (a) the pre-miR-M4 and (b) the mutant with the two nucleotide changes in the seed region (shown in blue). The sequence of the mature miRNA strands is shown in red.

Having demonstrated that the miRNAs encoded in the cluster 1 are essential for the oncogenic potential of the virus, we wanted to refine our investigation to identify whether any single miRNA within this cluster plays a more important role in the oncogenic potential of the virus. Since MDV miR-M4 in this cluster is a functional ortholog of the oncogenic miR-155 [19] implicated in a number of neoplastic disorders [23], we decided to focus on this particular miRNA. For this, we used the modified pRB1B5 clone [30] to generate two additional mutant viruses where only the miR-M4 was made non-functional. These included the constructs miR-4-00, where both copies of the pre-miR-M4 sequences were deleted, and the miR-4-0-mu2, where one of the deleted copies was replaced with a mutant miR-M4 carrying a 2-nucleotide mutation in the seed sequence (Fig. 7). We have previously shown that this mutation in the seed region abolished miR-M4 function [19]. We also determined the complete sequence of the ∼178-kb genome of the miR-4-0-mu2 construct by deep sequencing and ruled out unintended mutations or rearrangements generated during the BAC manipulation. Revertant construct miR-4-0-R was also generated by restoring one copy of the wild type miR-M4 sequence. A summary of the miRNA expression profiles of these constructs are in Table 1. The mutant viruses showed similar growth kinetics in CEF further demonstrating that miR-M4 deletion did not affect replication *in vitro* (Fig. 4B).

**Figure 8:**
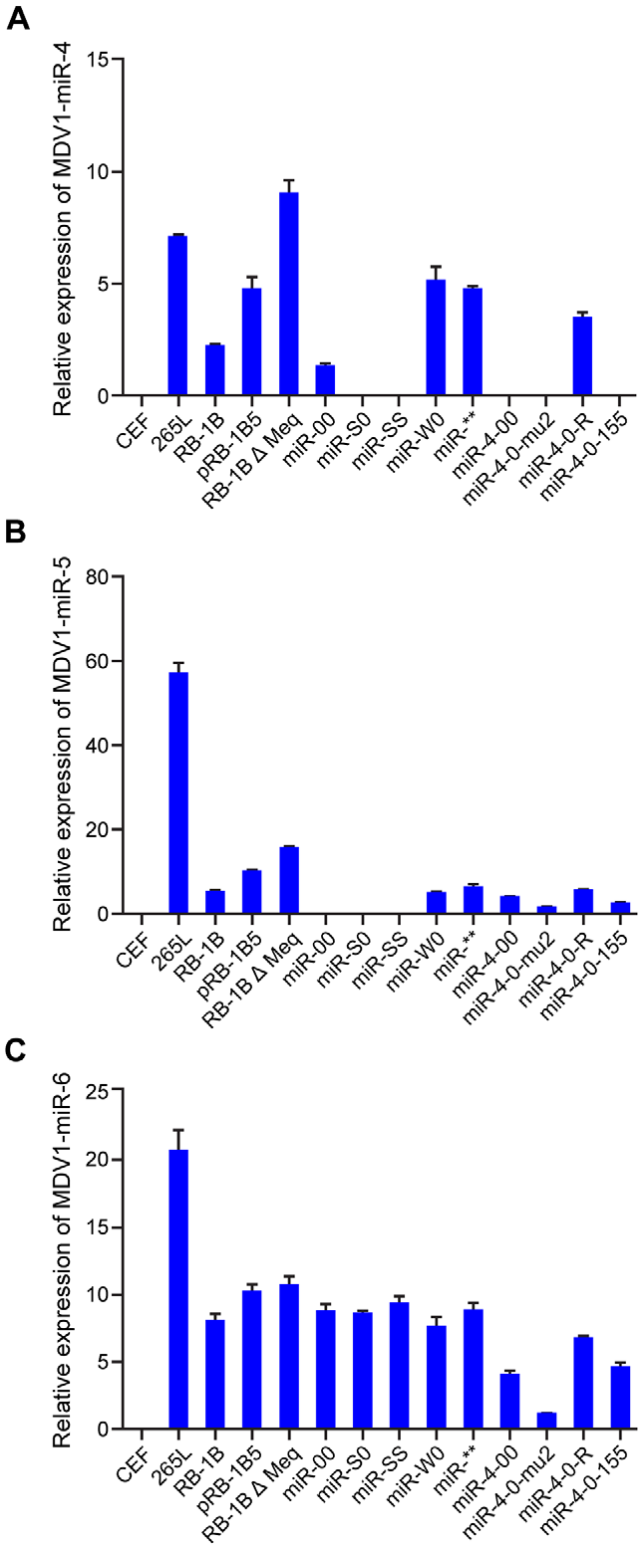
Expression of miR-M4, miR-M5 and miR-M6 in infected cells. Relative expression of (**A**) miR-M4, (**B**) miR-M5 and (**C**) miR-M6 measured by qRT-PCR in RNA extracted from CEF infected with the wild type RB-1B, the parent pRB-1B5 and each of the mutant viruses. Uninfected CEF, MDV-transformed lymphoblastoid cell line 265L and Meq-deletion mutant RB-1BΔMeq were used as controls. The lowest level of positive samples in each group (miR-00 for miR-M4, miR-4-0-mu2 for miR-M5 and miR-M6) was used as calibrator. Results represent the mean of triplicate assays with error bars showing the standard error of the mean.

We also examined the expression levels of MDV-encoded miRNAs miR-M4, miR-M5 and miR-M6 by qRT-PCR on RNA extracted from CEF infected with wild type RB-1B, the parent pRB-1B5 and the different mutant viruses (Fig. 8A–C). MDV-transformed cell line 265L and RB-1BΔMeq virus were used as controls. The absence of miR-M4 and miR-M5 transcripts in the cells infected by miR-S0 and miR-SS viruses (the low level of miR-M4 but not miR-M5 in miR-00 virus is due to the presence of one copy of miR-M4), confirmed the deletion of these miRNAs in these constructs (Fig. 8A,B). As expected, the expression of miR-M6 located within the LAT region was not affected by the mutations within the cluster 1 miRNA locus (Fig. 8C).

When the oncogenicity of these viruses was evaluated in MD lymphoma model in the natural chicken hosts, the parent pRB-1B5 virus induced 100 percent disease (Fig. 6B). Compared to this, none of the birds infected with miR-4-0mu2 virus showed any clinical evidence, gross tumors or microscopic lesions in any of the organs. There was 90% reduction of MD incidence in birds infected with miR-4-00 virus (Fig. 6B), clearly demonstrating the important role of miR-M4 in inducing lymphomas. The rescue of the oncogenic phenotype in 70% of birds by the revertant miR-4-0R virus even by restoring one copy of the wild type of miR-M4, further confirmed the significant role of miR-M4 in the induction of lymphomas.

### Oncogenic function of miR-M4-deleted virus can be rescued by gga-miR-155

**Figure 9:**
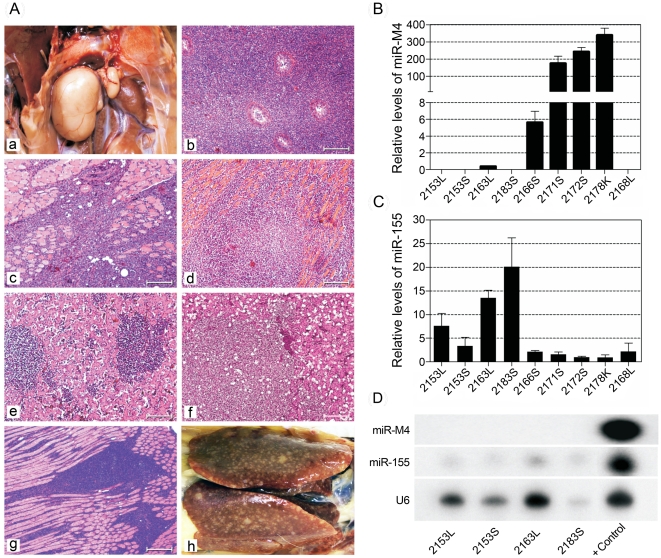
Pathological lesions and expression of miRNAs in tumors induced by MDV. (**A**) Pathology of MD lymphomas induced by different viruses in infected birds: Gross lesion showing lymphoma of the right testis (a) and histological lesions of lymphoma of the testis (b) and skeletal muscle (c) of birds infected with the parent pRB-1B5 virus; histological lesions of lymphomas in the cardiac muscle (d), and liver (e) in a bird infected with miR** virus; histological lesions of lymphoma of the liver (f) and skeletal muscle (g) in birds infected with miR-4-0-R virus; Gross lesions of the liver showing lymphomas (h) in the bird (#2163) infected with miR-4-0-155 virus. (Haematoxylin & Eosin stain, Bar = 50µM). (**B**) Relative expression of miR-M4 (normalised with the levels of miR-M8) in RNA extracted from tumor tissues induced by miR-4-0-155 (samples 2153L, 2153S, 2163L and 2183S), miR-4-0-R (samples 2166S, 2171S, 2172S and 2178K) and miR-4-00 (sample 2168L). As expected, none of the tumors from birds infected with miR-4-0-155 virus showed miR-M4 expression, while all the miR-4-0-R virus-induced tumors did show high levels of miR-M4 expression. (**C**) Relative expression of miR-155 (normalised to the let-7) in the above tumor tissues showed increased levels of miR-155 in the miR-M4-0-155 virus-infected tumors, but not in any of the miR-M4-0R virus-infected tumor tissues. No expression of miR-M4 but very low miR-155 was detected in the tumor that was induced in a single bird (2168L) infected with miR-M4-00 virus. (**D**) Confirmation of the expression of miR-155, but not miR-M4, in the miR-M4-0-155 virus-infected tumor tissues of samples by Northern blot analysis. The cellular U6 small nuclear RNA served as a loading control.

Since miR-M4 is a functional ortholog of the host-encoded gga-miR-155 [19], we wanted to examine whether gga-miR-155 can replace the oncogenic function of miR-M4 in MDV. For this, we generated an additional MDV construct miR-4-0-155 (Table 1), in which gga-miR-155 pre-miRNA including the loop sequence of the chicken BIC transcript [32] was introduced in the position of the viral miR-M4 pre-miRNA. After demonstrating that the *in vitro* growth kinetics on CEF was similar (Fig. 4B), we examined the oncogenic potential of miR-4-0-155 virus in the MD lymphoma models in chickens. Demonstration of the incidence of MD at similar levels (70%) in birds infected with miR-4-0-155 virus and the miR-4-0R revertant virus (Fig. 6B) indicated that gga-miR-155 can function as an oncogenic miRNA in the context of the MDV cluster 1 miRNAs. Although the levels of MD incidence were similar, the onset of tumors induced by miR-4-0-155 virus was slow, with most of the tumors detected much later than those induced by the parent pRB-1B5 or the miR-4-0R revertant viruses (Fig. 6B). Tumours induced by the mutant viruses, including the chimeric miR-4-0-155 virus, were typical MD lymphomas with neoplastic lymphoid lesions in multiple organs (Fig. 9A). In order to demonstrate the expression of gga-miR-155 or miR-M4 in the tumors induced by miR-4-0-155 and miR-4-0-R viruses respectively, we carried out quantitative RT-PCR on RNA samples extracted from the lymphomas collected from infected birds at *post mortem*. Demonstration of expression of miR-M4, but not gga-miR-155, in lymphoid tumors harvested from birds infected with miR-4-0-R virus (bird numbers #2166S, 2171S, 2172S and 2178K) indicated the direct correlation between the expression of miR-M4 and the induction of lymphomas (Fig. 9B–C). Despite the low levels of expression, detection of gga-miR-155 but not miR-M4 in tumors of birds infected with miR-4-0-155 virus (bird numbers #2153L, 2153S, 2163L and 2183S) by quantitative RT-PCR and Northern blotting (Fig. 9D) suggested a direct role for gga-miR-155 in induction of these tumors.

### Attenuated MDV deleted in miRNAs can serve as effective vaccine

**Figure 10:**
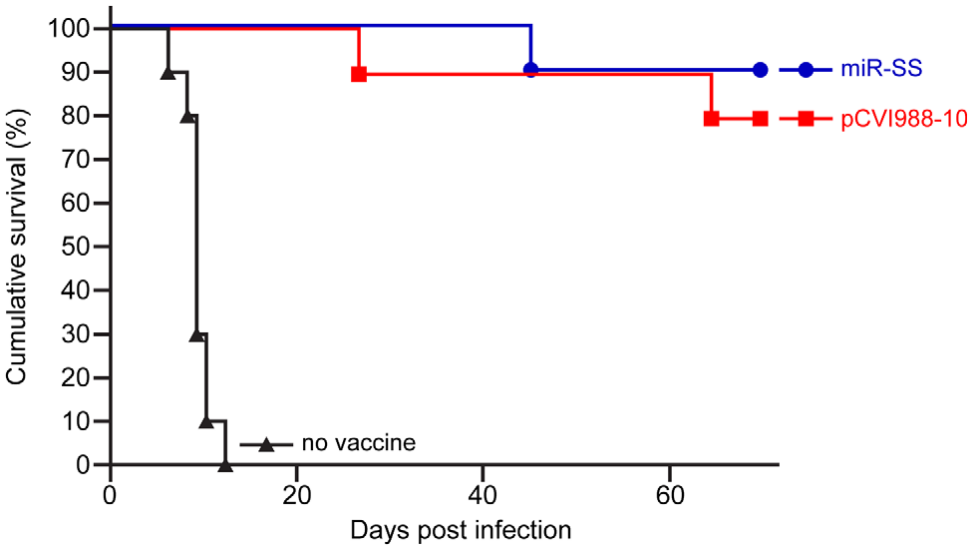
Protection from virulent MDV infection. Percentage of survival of unvaccinated or birds vaccinated with miR-SS or pCVI988-10 viruses after infection with vv+MDV strain 675A.

Demonstration of the total loss of oncogenicity persuaded us to investigate whether MDV made non-oncogenic by inactivation of miRNAs can function as effective vaccines against virulent MDV infection. For this, groups of birds were vaccinated with a recombinant (pCVI988-10) clone [33] of the widely used CVI988 vaccine strain or miR-SS virus that has mutations in all the miRNAs encoded from both copies of the cluster 1. Birds from all the groups were challenged with the very virulent plus MDV (vv+MDV) strain 675A along with unvaccinated controls. Comparison of the incidence of MD in the 3 groups during the 68-day experimental period showed that miR-SS virus provided the same level of protection as the widely used Rispens vaccine against infection by the vv+MDV strain 675A that induced 100 per cent disease in unvaccinated chickens (Fig. 10).

## Discussion

Despite the increasing evidence of the potential role of herpesvirus-encoded miRNAs functioning as determinants of oncogenicity [34], direct role of any of these miRNAs in the induction of tumors *in vivo* has not yet been demonstrated. Using lymphoma models in the natural chicken hosts infected with modified recombinant viruses generated from infectious BAC clones of the oncogenic RB-1B strain of MDV, our study provides the first direct evidence of the role of miRNAs in the induction of tumors. The total abolition of tumors in birds infected by viruses that do not express miRNAs in cluster 1, and subsequent rescue of the oncogenic phenotype by the revertant miR-W0 virus demonstrated the direct role of these miRNAs in the induction of MD lymphomas. Moreover, the inability of miR-S0 and miR-SS viruses to induce lymphomas and restoration of the oncogenic phenotype by miR** virus revealed that the miRNAs, and not the overlapping R-LORF8 gene, are the major determinants of oncogenicity. Important role of these miRNAs in oncogenesis is supported by the high levels of their expression in MD tumors and transformed cell lines [16], [18].

Although other miRNAs encoded within this cluster might be contributing to the induction of MD lymphomas [35], we focussed mainly on examining the role of miR-M4 for various reasons. Firstly, MDV miR-M4 is expressed at very high levels in lymphomas, in some cases accounting for even up to 72% of all MDV-encoded miRNAs [16], [17], [18], supporting its potential as a major determinant of MDV oncogenicity. Secondly, based on the seed sequence homology and the potential for regulating common targets such as Pu.1, BACH1, CEBPβ, HIVEP2, BCL2L13 and PDCD6, we have previously shown that miR-M4 is a functional ortholog of gga-miR-155 [19], a miRNA known to be directly associated with several cancers [23], [36] and molecular mechanisms of cancer pathogenesis [25], [26]. Finally, overexpression of miR-155 has been shown to be associated with lymphocyte transformation by viruses such as EBV [37] and reticuloendotheliosis virus strain T [38]. Significant reduction in the incidence of MD in birds infected with miR-4-00 virus, and the total loss of oncogenicity of miR-4-0-mu2 virus clearly indicated that miR-M4 plays a critical role in the induction of MD lymphomas. Although we have not carried out similar investigations on other miRNAs in this cluster, the dramatic suppression of oncogenicity subsequent to the loss of miR-M4 function to the levels similar to those shown by viruses with mutations in the whole cluster 1 miRNAs, suggested that miR-M4 is very important for the oncogenicity of MDV. However the expression of miR-M4 alone appeared to be not sufficient since miR-00 virus expressing low levels of miR-M4 (Fig. 8A) remained non-oncogenic, suggesting the contribution of other miRNAs in the cluster to the oncogenicity. This is also supported by our recent observation that viruses deleted in miR-M5, another miRNA within this cluster, retained oncogenicity although not to the same extent as the wild type viruses (unpublished data). The exact mechanisms of the loss of oncogenic phenotype by the miR-M4-deleted/mutated viruses remain to be investigated. However, it is not due to the lack of expression of viral genes such as Meq, ICP4 and LAT as we were able to demonstrate the expression of the proteins or transcripts in infected CEF or PBL (Fig. 3 and 5). It is also not due to the inability to express miRNAs such as miR-6 outside the cluster 1 (Fig. 8C). MDV miR-M4 is known to influence directly the expression of a number of viral and cellular targets [19], [22]. The regulation of gene expression by these miRNAs can also be due to indirect effects, and may include genes such as Meq. Although the expression levels of Meq in CEF infected with miR-M4-deleted/mutated viruses were not affected (Fig. 5B), the relative levels of Meq in the PBL of infected birds were lower than those from birds infected with viruses expressing the miRNAs (Fig. 5C). Meq is well-known for its key role in MDV oncogenesis [11]–[15]. Although Meq by itself is weak in its oncogenic potential, modulation of the levels of Meq expression by these miRNAs could be important in MDV oncogenicity. Our recent studies have shown that Meq may have a regulatory role in the expression of cluster 1 miRNAs by binding to its promoter region (unpublished) suggesting that Meq-miRNA regulatory loop could be important in the induction of tumors. Thus viral oncogenesis should be seen as a very complex process involving the interaction of multiple factors and regulatory processes including those by the virus-encoded miRNAs. Disruption of any one of these key elements could have a significant effect on the oncogenic pathways. Although the precise understanding of all the molecular processes would need further studies, the present findings demonstrate that miR-M4, most likely acting through the miR-155 pathway, functions as a key factor contributing to MD oncogenesis.

It is remarkable to show that a two-nucleotide mutation in the miR-M4 seed sequence in the context of the whole viral genome was sufficient for the total inhibition of oncogenicity of the miR-4-0-mu2 virus (Fig. 6B). One of the birds (#2168L) infected with miR-4-00 virus did develop MD lymphoma even in the absence of miR-M4 (Fig. 9B, C). This is unlikely to be due to any spurious mutations or recombination events in the virus, as we were able to confirm the absence of miR-M4 or miR-155 expression in the tumor tissues of this bird (Fig. 9D). We are yet to identify the exact mechanisms underlying the development of lymphomas in this bird in the absence of miR-M4. However, occurrence of such tumors underlines the complex and multifactorial nature of oncogenic processes; moreover the individual differences between birds in immunocompetence and genetic susceptibility could also contribute to the onset of such tumors. Nevertheless, the near total elimination of oncogenicity in the absence of miR-M4, and the rescue of oncogenicity in up to 70 per cent of the birds when the miR-M4 expression was restored in the revertant miR-4-0-R virus, provided the first clear evidence on the *in vivo* role of a virus-encoded miRNA in the induction of tumors.

The conservation of the seed sequence and the ability to regulate common sets of targets by miR-M4 and gga-miR-155 [19] prompted us to examine whether the miR-M4 functions of MDV can be replaced by gga-miR-155. The demonstration of the ability of the chimeric miR-4-0-155 virus to restore the oncogenic potential to the same levels as the miR-4-0-R (Fig. 6B), further demonstrated the significance of the miR-M4/miR-155 pathway in MD lymphomagenesis. Although the types of tumors induced by recombinant viruses expressing miR-M4 or gga-miR-155 were not distinguishable (Fig. 9A), the onset of tumors in birds infected miR-4-0-155 virus was slow (Fig. 6B), possibly due to the differences in the functional context of the two miRNAs or in their processing and expression. This was also evident from the expression levels of miR-M4 and miR-155 in the primary tumor samples collected from birds infected with the two viruses. Compared to the high levels of miR-M4 in tumor samples induced by miR-4-0-R virus (Fig. 9B), the levels of miR-155 in the tumors induced by miR-4-0-155 virus were much lower (Fig. 9B). The lack of expression of miR-M4 and weak expression of miR-155 in the tumors induced by miR-4-0-155 virus was also confirmed by Northern blot (Fig. 9D). Interestingly, there is consistent downregulation of gga-miR-155 in MD tumors and MDV-transformed cell lines [17], [18]. The downregulation of miR-155 in MDV-transformed tumor cell lines is not a permanent defect, as these cells can be induced to express miR-155 by co-infection with retroviruses-expressing v-Rel (unpublished data). Moreover, recent studies have demonstrated differences between miR-M4 and gga-miR-155 in targeting genes such as UL28 despite having identical seed sequences highlighting the significance of the non-seed regions of these miRNAs [22]. Tumors induced by the chimeric miR-4-0-155 virus are the first examples demonstrating the expression of gga-miR-155 in MD tumors. Although our study does not identify the various genes linked to the miR-M4-mediated oncogenesis, demonstration of the significance of miR-M4 in the induction of lymphomas is a major step in understanding the molecular oncogenic mechanisms in MD. From the demonstration of the conserved functions of miR-M4 and miR-155 in the induction of tumors, it is clear that MDV is able to restore the functions of miR-155 through the expression of high levels of the functional homolog miR-M4. Although it is unclear how MDV is able to downregulate miR-155, some feedback regulatory mechanisms are the most likely mechanisms. Interestingly in KSHV-induced tumors also, there is upregulation of the miR-155 functional homolog miR-K12-11, at the expense of significant downregulation of miR-155 [39]. On the other hand, EBV does not encode any functional ortholog but induces miR-155 in transformed B-cells [37], and a recent study has demonstrated its role in the induction of B-cell transformation by EBV *in vitro* [40]. It is unclear what advantages MDV and KSHV do have in choosing this rather complicated pathway of encoding and expressing high levels of the functional orthologs of a host miRNA, the expression of which is repressed in the transformed cells. One possible advantage of encoding a viral ortholog is the potential to achieve high levels of expression as seen in MDV- and KSHV-induced tumors, overriding the tighter cellular regulatory controls associated with the c-*bic*/miR-155 expression. Moreover, although miR-155 and the two viral orthologs may regulate common set of target proteins through the conserved seed sequences, other potential differences in their functions, especially due to the differences in the non-seed sequences, may also exist. It is not known whether the changes in the non-seed sequences do affect the functions of these miRNAs. Nevertheless, the differences in the speed in the onset of tumors between MDV expressing miR-M4 and gga-miR-155 would suggest that such differences could be important.

The role of miR-M4 and the rescue of the oncogenic phenotype by miR-155 provide further evidence of the conserved biological functions of miRNA orthologs, a finding of major importance in elucidating the functions of other viral orthologs such as KSHV miR-K12-11. The study also highlights the use of tumor virus disease models as powerful tools to reveal fundamental molecular determinants that trigger the development of cancers. Finally, the demonstration of protection induced by the miR-SS virus against infection by the vv+MDV strain 675A to the same levels as the widely used CVI988 vaccine strain (Fig. 10) demonstrated the prospects of generating molecularly-defined attenuated vaccines by specific deletion of oncogenic sequences such as the miRNAs.

## Materials and Methods

### Ethics statement

All animal experiments were performed in accordance with the United Kingdom Home Office guidelines under the provisions of the Project License approved by the Institute for Animal Health Ethical Committee.

### Cell culture and reconstitution of recombinant viruses

Primary chicken embryo fibroblast cultures (CEF) were prepared from 10-day old chicken embryos from SPF eggs as previously described [29]. Reconstitution of recombinant viruses was achieved by transfection of 1–2µg BAC DNA into the CEF using Lipofectamine (Invitrogen).

### Construction of viruses with deletion or mutations in cluster 1 miRNAs

Infectious BAC clone pRB-1B5 [29], [30] was used for the generation of the mutant constructs [41]. List of primers used for the construction are shown separately (Table S1). Selection markers *gal*K-kanamycin (*gal*K-Kn) [42] and thymidylate synthase-spectinomycin (*Thy*A-spec) [43] cassettes were used in consecutive steps for the deletion of the two copies of the miRNA cluster. For this, the fragment containing the miRNA cluster (GenBank EF523390 - Nucleotides 134362 to 136848) was amplified with 5′ GCCAACTGTACACGCAGGGACGT 3′ and 5′ GTGCAGTGCCTTTGATGTCTG 3′ primers and cloned into pCR8-TOPO vector (Invitrogen). From this vector, the 1665-bp *Psh*AI-*Stu*I fragment (134527–136183) encompassing all the six miRNAs in the cluster 1 from miR-M9 to miR-M4 (Fig. 1) was replaced with *gal*K-Kn cassette to generate the -recombination –construct for replacing the first copy of the microRNA cluster 1. Similarly, an *Xho*I fragment (135221–135959) from this vector was replaced with a *Thy*A-spec cassette to make another construct for the specific replacement of the second copy of the miRNA cluster. This shorter (783-bp) deletion removed all the miRNAs except miR-M9 and miR-M4. The two copies of the miRNA cluster were sequentially deleted using recombineering techniques [41], [44] to generate the miR-00 construct. For generation of viruses with mutations in all of the miRNAs within the cluster, a 1.45 kb *Ngo*MIV-*Eco*RV fragment corresponding to the positions 134785–136216 of the pRB-1B5 sequence was synthesised. The synthetic gene was designed through alternative codon usage so as to destroy all the pre-miRNA's hairpin structures, but retaining the R-LORF8 open reading frame (Fig. 2). The first copy of the miRNA cluster 1 was replaced using a RecA-based strategy [28]. Recombinant clones detected by PCR (primers 5′ GTAGTGTATCGGTCTTCGTG 3′ and 5′ CCCGAATACAAGGAATCCTG 3′) were digested with *Bgl*II to identify the clones with the replaced synthetic sequence that contained a unique *Bgl*II site. The virus reconstituted from this construct with one copy of the miRNA cluster replaced with the synthetic sequence and the other copy deleted by the insertion of the *gal*K-kn was designated miR-S0. The *gal*K-kn selection marker in the miR-S0 construct was then replaced with the pKOV-miR-syn construct to generate the construct with both copies of the miRNA cluster 1 replaced by synthetic sequences, and named miR-SS. We also generated a revertant construct miR-W0, in which we replaced one copy of wild type miRNA cluster sequence from pRB-1B5, while the other copy remains deleted. We mutated one copy of R-LORF8 start codon by inserting FRT-Kn cassette amplified by PCR using R-LORF8-Kn-For and R-LORF8-Kn-Rev primers (Table S1). The FRT-Kn cassette was ‘Flipped-off’ in *E coli* strain SW105, leaving only a ‘scar’ sequence. In order to prevent the translation of R-LORF8, we introduced stop codon replacing the ATG initiation codon in the construct miR**. For this, we first destroyed the gene by introducing the *gal*K-kn cassette into the locus. This was then replaced with a modified R-LORF8 sequence generated by PCR using R-LORF8-stop-For and R-LORF8-stop-Rev primers (Table S1). Detailed protocols for the manipulation of the BAC constructs are provided separately [41]. The accuracy of the mutations in different constructs was checked by sequencing.

### Construction of viruses with deletion or mutations of miR-M4

In order to examine the role of miR-M4 in MDV oncogenicity, we also constructed a series of viruses with mutations only in the miR-M4, by standard mutagenesis techniques [45] on the pRB-1B5 clone [29]. First we deleted one copy of the pre-miR-M4 with FRT-Kn cassette using PCR product derived with miR-M4-kn-For and miR-M4-kn-Rev primers. After excision of the Kn cassette, the second copy was deleted with *gal*K cassette using PCR products derived with miR-M4-*gal*K-For and miR-M4-*gal*K-Rev primers. This construct with deletion of both copies of the miR-M4 was designated miR-M4-00 and the *gal*K locus in this construct was used for generating additional constructs. For the construction of a seed mutant of miR-M4, we generated a PCR product with miR-M4-mu2-Top and miR-M4-mu2-Bottom primers, designed to introduce two-nucleotide mutations in the miR-M4 seed region that has been previously shown to be sufficient to abolish the function of miR-M4. The *gal*K selection marker in the miR-M4-00 construct was replaced with the mutated miR-M4 to generate the miR-4-0-mu2 construct. In order to generate another construct in which the miR-M4 was replaced with the miR-155 sequence, we generated the gga-miR-155 pre-miRNA along with the loop sequence using miR-M4-155-Top and miR-M4-155-Bottom primers. The *gal*K selection marker in the miR-4-00 construct was replaced with the annealed above oligonucleotides to generate the miR-4-0-155. Finally, the revertant construct miR-4-0-R was generated in which the wild type miR-M4 sequence was restored. The accuracy of all the modifications was also confirmed by sequencing the modified regions of the constructs. The whole genome of the miR-4-0-mu2 was determined by deep sequencing to confirm the mutations and to rule out any unexpected mutations or recombinations.

### Virus infection studies *in vivo*

All animal experiments were carried out under licence from the UK Home Office in dedicated negative pressure rooms. Groups (n = 10–12) of one day-old line P (MHC type B^19^/B^19^) chicks were infected with 1,000 plaque forming units (pfu) of miR-00, miR-S0, miR-SS, miR-W0, miR**, miR-4-00, miR-4-0-mu2, miR-4-0-155 and miR-4-0-R and pRB1B5 viruses as described. Blood samples collected at regular intervals or post-infection (d. p. i.) were used for quantitation of virus load or for the extraction of RNA from the PBL. Methods for quantitative RT-PCR to measure miRNA/transcript levels have been described [31]. Virus isolations were also performed by inoculating 1×10^6^ PBL to a well of a 6-well plate of CEF and incubated at 38.5 C in 5% CO_2_ until the appearance of virus plaques. The birds were inspected regularly and were sacrificed at clinical end-points and samples taken post-mortem for histology. Birds showing gross or histological lesions of lymphomas in any of the tissues collected at post-mortem were diagnosed as MD-positive, and the data from the incidence of MD from each of the groups were used to calculate the cumulative survival rates.

### Vaccination and challenge studies

Groups (n = 10) of one-day old birds were vaccinated via the intra-muscular route with either sham vaccine, or 925 pfu of miR-SS or pCVI988 viruses. One week after infection, all the birds were infected with 1450 pfu of the vv+MDV strain 675A via the intra-peritoneal route. Birds were observed for the onset of clinical disease and the incidence of MD was recorded up to 68 days post infection. All the birds were necropsied at the end of the experiment and the incidence of MD from the gross and microscopic lesions was used to calculate the survival rates from virus infection.

### Detection of viral antigens

Western blotting to detect the viral proteins Meq, pp38 and pp14, and the chicken CtBP1 was carried out using methods previously described [11]. Immunofluorescence staining and confocal microscopy were carried out on CEF cultures on 13 mm glass coverslips infected with the mutant viruses using methods described [12]. The infected cell were fixed in 4% paraformaldehyde, permeabilised with 0.1% Triton x-100 and stained with anti-Meq antibody (FD7) or anti-pp38 (BD1) antibodies and detected with Alexa Fluor 488/568-conjugated goat anti-mouse reagents (Invitrogen). Images were taken using Leica TCS SP5 confocal laser scanning microscope.

## Supporting Information

Table S1 List of oligonucleotides used in the experiments.
(0.04 MB DOC)
