# Supplementary material for: Critical Role of the Virus-Encoded MicroRNA-155 Ortholog in the Induction of Marek's Disease Lymphomas
Source: PLoS Pathog. 2011 Feb 24;7(2):e1001305. doi: 10.1371/journal.ppat.1001305 (PMC3044692; doi:10.1371/journal.ppat.1001305)
Supplement: Table S1 — List of oligonucleotides used in the experiments. (0.04 MB DOC) [file ppat.1001305.s001.doc]

**Table S1 Sequences of oligonucleotides used in the mutagenesis**

| Name | Sequence |
| --- | --- |
| R-LORF8-Kn-For | 5' CTCCGTATCA CTCCCGAACCATTAGATATCAGTCGATCCAGCCCCCCACGTCATGTGTAGGCTGGAGCTGCTTC 3' |
| R-LORF8-Kn-Rev | 5' GTGATGTAATATTGAATCGGATTTGGAATAACCGAATTCGGTGATATAAAGACGATAGATTCCGGGATCCGTCGAC 3' |
| R-LORF8-stop-For | 5' CGTCATGCAAGCTTAATAATAACTATCGTCTTTATATCACC 3' |
| R-LORF8-stop-Rev | 5' GACGATAGTTATTATTAAGCTTGCATGACGTGGGGGGCTGGA 3' |
| miR-M4-kn-For | 5' GACGTGTTCCACGTGACGGCTCTGGGCTTGTTTTGAATGTCCCCTTGAGTTGTGTAGGCTGGAGCTGCTTC 3' |
| miR-M4-kn-Rev | 5' GGTCATGCTGTCAGAACCATTCGTGGTCACCGAACGAAGGGTTCCGATACATTCCGGGGATCCGTCGAC 3' |
| miR-M4-*gal*K-For | 5' GACGTGTTCCACGTGACGGCTCTGGGCTTGTTTTGAATGTCCCCTGTGAAGTTCTGTTGACAATTAATCATCGGCA 3' |
| miR-M4-*gal*K-Rev | 5' TCACTTCAACGGTGTATAGACGTTCTACGATGGTTTTCCAGCGATCATTCCAGCACTGTCCTGCTCCTT 3' |
| miR-M4-mu2-Top | 5' CTTGTTTTGAATGTCCCCTGTGAAGTTTATTCCTGTATCGGAACCCTTCGTTCGGTGACCACGAATGGTTCTGACAGGAAGACCTTTCTGGGGGAAATGATCGCTGGAAA 3' |
| miR-M4-mu2-Bottom | 5' TTTCCAGCGATCATTTCCCCCAGAAAGGTCTTCCTGTCAGAACCATTCGTGGTCACCGAACGAAGGGTTCCGATACAGGAATAAACTTCACAGGGGACATTCAAAACAAG 3' |
| MiR-M4-155-Top | 5' GAATGTCCCCTGTGAAGTTTAATGCTAATCGTGATAGGGGTTTTTACCTCTGAATGACTCCTACATGTTAGCATAACATTCTGGGGGAAATGATCGCTGGAAACCATCG 3' |
| MiR-M4-155-Bottom | 5' CGATGGTTTCCAGCGATCATTTCCCCCAGAATGTTAATGCTAACATGTAGGAGTCATTCAGAGGTAAAAACCCCTATCACGATTAGCATTAAACTTCACAGGGGACATTC 3' |
